# Supplementary material for: Divergent Regulation of Myotube Formation and Gene Expression by E2 and EPA during In-Vitro Differentiation of C2C12 Myoblasts
Source: Int J Mol Sci. 2020 Jan 23;21(3):745. doi: 10.3390/ijms21030745 (PMC7037418; doi:10.3390/ijms21030745)
Supplement: Supplementary file 1 [file ijms-21-00745-s001.pdf]

Table S1: Gene ontology (GO) of up and down regulated genes for paired analysis of E2 vs Con-Ve; EPA vs Con-Ve and EPA vs E2

**GO E2 vs Con-Ve increased expression**			**GO E2 vs Con-Ve decreased expression**			**GO EPA vs Con-Ve increased expression**			**GO EPA vs Con-Ve decreased expression**			**GO EPA vs E2 increased expression**			**GO EPA vs E2 decreased expression**		
**Term**	**No**	**FC (Log)**	**Term**	**No**	**FC (Log)**	**Term**	**No**	**FC (Log)**	**Term**	**No**	**FC (Log)**	**Term**	**No**	**Log2 FC**	**Term**	**No**	**FC (Log)**
myosin filament	9	5.98	MHC class Ib protein complex	2	5.41	CHOP-C/EBP complex	3	4.51	troponin complex	8	4.18	TAP complex	3	3.78	troponin complex	8	4.13
muscle myosin complex	4	5.93	symbiont-containing vacuole membrane	4	5.00	extrinsic component of external side of plasma membrane	4	3.51	junctional sarcoplasmic reticulum membrane	3	4.18	MHC class I protein complex	9	3.37	junctional sarcoplasmic reticulum membrane	3	4.13
sarcoplasmic reticulum lumen	3	5.78	high-density lipoprotein particle	4	3.60	IPAF inflammaso me complex	3	3.51	muscle myosin complex	5	3.92	symbiont-containing vacuole membrane	4	3.20	striated muscle thin filament	18	3.78
cardiac myofibril	2	5.51	very-low-density lipoprotein particle	3	3.33	HFE-transferrin receptor complex	3	3.51	myosin filament	10	3.81	extrinsic component of external side of plasma membrane	4	3.20	myofilament	19	3.68
myofibril	12	4.78	phagocytic vesicle membrane	5	2.76	MHC class I protein complex	5	2.83	laminin-5 complex	3	3.77	IPAF inflammaso me complex	3	3.20	myosin filament	13	3.66
interstitial matrix	5	4.75	blood microparticle	11	2.40	very-low-density lipoprotein particle	5	2.58	striated muscle thin filament	10	3.70	Golgi medial cisterna	7	2.92	muscle myosin complex	6	3.54
I band	7	4.74	apical part of cell	6	1.74	Golgi lumen	5	2.44	junctional membrane complex	5	3.51	endoplasmic reticulum exit site	7	2.76	junctional membrane complex	5	3.45
striated muscle thin filament	4	4.71	extracellular space	67	1.51	high-density lipoprotein particle	5	2.44	muscle tendon junction	3	3.45	integral component of lumenal side of endoplasmic reticulum membrane	5	2.61	terminal web	3	3.39
sarcomere	8	4.31	extracellular region	77	1.49	interstitial matrix	4	2.42	terminal web	3	3.45	phagocytic vesicle membrane	13	2.34	sarcoplasmic reticulum lumen	3	3.39
myosin complex	10	4.14	proteinaceous extracellular matrix	13	1.39	Golgi medial cisterna	4	2.42	sarcoplasmic reticulum lumen	3	3.45	integral component of nuclear inner	4	2.29	unconventional myosin complex	3	3.39

												membrane					
contractile fiber	3	3.85	extracellular matrix	11	1.26	endoplasmic reticulum exit site	4	2.26	myosin II complex	4	3.18	very-low-density lipoprotein particle	5	2.27	muscle tendon junction	3	3.39
M band	3	3.64	cell surface	22	1.16	vacuole	5	2.25	I band	12	3.18	high-density lipoprotein particle	5	2.13	myosin II complex	10	3.36
sarcoplasmic reticulum membrane	4	3.47	external side of plasma membrane	11	1.15	phagocytic vesicle membrane	8	1.95	actomyosin contractile ring	19	3.18	extracellular matrix	43	1.42	collagen network	3	3.13
sarcoplasmic reticulum	7	3.46	endoplasmic reticulum membrane	23	1.05	extracellular vesicle	8	1.89	myofibril	19	3.11	microvillus	11	1.41	actomyosin contractile ring	3	3.13
A band	3	3.46	neuronal cell body	15	0.84	proteinaceous extracellular matrix	46	1.73	contractile fiber	9	3.11	extracellular vesicle	7	1.39	collagen type IV trimer	3	3.13
T-tubule	6	3.27	endoplasmic reticulum	36	0.80	anchored component of membrane	20	1.69	fascia adherens	6	3.07	external side of plasma membrane	44	1.35	network-forming collagen trimer	3	3.13
Z disc	13	3.26	cytoplasmic vesicle	17	0.75	extracellular matrix	41	1.67	semaphorin receptor complex	4	3.02	lipid particle	9	1.32	basement membrane collagen trimer	3	3.13
intercalated disc	5	3.16	membrane	131	0.26	external side of plasma membrane	42	1.59	sarcomere	16	2.98	proteinaceous extracellular matrix	42	1.29	fascia adherens	6	3.01
chloride channel complex	4	2.99				extracellular space	178	1.43	M band	9	2.90	apical part of cell	15	1.26	sarcoplasmic reticulum membrane	16	2.96
proteinaceous extracellular matrix	24	2.80				extracellular region	204	1.41	sodium:potassium-exchanging ATPase complex	4	2.86	lysosome	43	1.25	semaphorin receptor complex	4	2.96
stress fiber	5	2.77				melanosome	11	1.31	Z disc	48	2.82	extracellular space	195	1.25	contractile ring	5	2.87
basement membrane	6	2.50				collagen trimer	9	1.30	dystrophin-associated glycoprotein complex	5	2.81	caveola	11	1.23	glycoprotein complex	7	2.85
sarcolemma	7	2.44				organelle membrane	10	1.28	costamere	7	2.74	anchored component of membrane	18	1.23	dystrophin-associated glycoprotein complex	7	2.85
actin filament	4	2.43				basement membrane	10	1.23	flotillin complex	4	2.73	extracellular region	75	1.20	M band	9	2.84

actin cytoskeleton	8	1.86				cytoplasmic, membrane-bounded vesicle	15	1.18	actomyosin	4	2.73	cell surface	75	1.13	myofibril	86	2.78
apical plasma membrane	10	1.48				secretory granule	11	1.16	voltage-gated sodium channel complex	5	2.70	melanosome	12	1.13	I band	54	2.78
cell surface	16	1.22				lysosome	31	1.09	interstitial matrix	6	2.68	organelle membrane	11	1.10	contractile fiber part	81	2.77
extracellular region	41	1.10				cell surface	57	1.04	myosin complex	18	2.65	perikaryon	16	0.97	contractile fiber	89	2.77
extracellular space	28	0.77				blood microparticle	12	1.04	stress fiber	23	2.64	cytoplasmic, membrane-bounded vesicle	16	0.96	Z disc	48	2.76
extracellular exosome	42	0.52				perikaryon	13	0.98	intercalated disc	17	2.60	blood microparticle	14	0.95	sarcomere	76	2.75
cytoplasm	92	0.34				neuronal cell body	41	0.81	sarcoplasmic reticulum membrane	11	2.60	membrane raft	24	0.75	myosin complex	25	2.75
membrane	92	0.27				neuron projection	32	0.79	plasma membrane raft	4	2.60	lysosomal membrane	21	0.72	sarcoplasmic reticulum	24	2.71
						axon	27	0.73	sarcoplasmic reticulum	19	2.57	extracellular exosome	234	0.68	costamere	7	2.69
						extracellular exosome	194	0.72	neuromuscular junction	20	2.51	neuronal cell body	45	0.63	complex of collagen trimers	7	2.69
						intracellular membrane-bounded organelle	48	0.54	(T-tubule	16	2.35	intracellular membrane-bounded organelle	63	0.62	flotillin complex	4	2.67
						cytosol	104	0.41	A band	7	2.35	axon	31	0.62	neuromuscular junction	23	2.65
						endoplasmic reticulum	77	0.41	integrin complex	7	2.35	endoplasmic reticulum	107	0.57	voltage-gated sodium channel complex	5	2.64
						integral component of plasma membrane	65	0.39	lamellipodium membrane	5	2.26	cytoplasmic vesicle	51	0.54	interstitial matrix	6	2.63
						mitochondrion	93	0.30	sarcolemma	30	2.21	cytosol	139	0.48	sarcoplasm	26	2.62
						membrane	343	0.16	axonal growth cone	6	1.96	integral component of plasma membrane	84	0.45	A band	15	2.54
									basement membrane	20	1.91	endoplasmic reticulum membrane	52	0.43	integrin complex	10	2.54

									presynaptic active zone	6	1.86	Golgi apparatus	86	0.41	banded collagen fibril	4	2.54
									basal plasma membrane	8	1.79	perinuclear region of cytoplasm	49	0.38	fibrillar collagen trimer	4	2.54
									cortical actin cytoskeleton	8	1.76	membrane	432	0.18	protein complex involved in cell adhesion	10	2.41
									actin cytoskeleton	36	1.70	cytoplasm	406	0.17	actomyosin	23	2.40
									cytoplasmic side of plasma membrane	8	1.63				stress fiber	20	2.38
									filopodium	13	1.62				contractile actin filament bundle	20	2.38
									lateral plasma membrane	9	1.60				sodium channel complex	5	2.36
									Glamellipodium	25	1.47				intercalated disc	16	2.32
									presynapse	9	1.38				T-tubule	17	2.31
									proteinaceous extracellular matrix	43	1.31				filopodium membrane	5	2.28
									actin filament	9	1.27				actin filament bundle	20	2.24
									receptor complex	17	1.21				basement membrane	25	2.09
									growth cone	20	1.19				sarcolemma	36	2.04
									postsynaptic density	30	1.19				cell-cell contact zone	17	2.03
									endomembrane system	14	1.16				extracellular matrix component	31	2.02
									bicellular tight junction	16	1.15				cortical actin cytoskeleton	15	2.01
									basolateral plasma membrane	24	1.10				actin cytoskeleton	101	1.95
									cytoplasmic vesicle membrane	14	1.09				filopodium	20	1.79
									cell junction	83	1.07				cortical cytoskeleton	18	1.74
									focal adhesion	45	1.07				lamellipodium	32	1.68
									cytoskeleton	119	0.96				presynaptic active zone	6	1.63
									axon	39	0.94				proteinaceous extracellular matrix	63	1.61

									cell cortex	15	0.92				cell division site part	10	1.59
									cell surface	62	0.84				cell division site	10	1.59
									cell-cell junction	19	0.78				collagen trimer	15	1.56
									apical plasma membrane	31	0.78				lateral plasma membrane	9	1.54
									cell-cell adherens junction	29	0.74				basal plasma membrane	7	1.54
									microtubule	30	0.74				cell cortex part	22	1.53
									postsynaptic membrane	20	0.71				kinesin complex	9	1.52
									synapse	45	0.70				cell surface furrow	8	1.51
									perinuclear region of cytoplasm	60	0.66				cleavage furrow	8	1.51
									dendrite	42	0.64				plasma membrane raft	17	1.49
									neuronal cell body	45	0.62				actin filament	16	1.43
									integral component of plasma membrane	91	0.56				cell cortex	38	1.35
									Golgi membrane	32	0.53				caveola	14	1.35
									endoplasmic reticulum	103	0.50				basal part of cell	9	1.32
									endoplasmic reticulum membrane	54	0.47				postsynaptic specialization	34	1.32
									cell projection	54	0.46				postsynaptic density	34	1.32
									cytoplasmic vesicle	48	0.43				transcriptional repressor complex	11	1.28
									cytoplasm	490	0.43				cell leading edge	52	1.27
									membrane	507	0.40				actin-based cell projection	29	1.27
									cytosol	122	0.31				apical junction complex	20	1.24
									plasma membrane	332	0.31				excitatory synapse	35	1.24
									extracellular exosome	172	0.23				extracellular matrix	69	1.21
															cytoplasmic region	41	1.19

															ruffle	21	1.18
															cell-substrate adherens junction	50	1.14
															receptor complex	41	1.14
															site of polarized growth	24	1.14
															cell-substrate junction	50	1.13
															growth cone	23	1.11
															dendritic spine	19	1.11
															neuron spine	19	1.09
															focal adhesion	47	1.07
															synapse	104	1.05
															postsynapse	53	1.05
															basolateral plasma membrane	27	1.01
															bicellular tight junction	15	1.00
															synapse part	79	0.99
															terminal bouton	13	0.98
															occluding junction	15	0.97
															apical plasma membrane	37	0.97
															postsynaptic membrane	25	0.97
															adherens junction	77	0.97
															anchoring junction	79	0.96
															cytoskeleton	225	0.95
															cell-cell adherens junction	36	0.94
															plasma membrane receptor complex	18	0.94
															cell junction	154	0.94
															synaptic membrane	33	0.93

															leading edge membrane	16	0.93
															cation channel complex	17	0.92
															integral component of endoplasmic reticulum membrane	14	0.91
															cell-cell junction	73	0.90
															apical part of cell	45	0.90
															plasma membrane region	106	0.88
															neuron projection terminus	21	0.86
															plasma membrane protein complex	50	0.86
															intrinsic component of endoplasmic reticulum membrane	14	0.86
															cytoskeletal part	160	0.86
															transmembrane transporter complex	30	0.84
															ion channel complex	27	0.82
															axon terminus	19	0.82
															transporter complex	30	0.81
															perinuclear region of cytoplasm	71	0.81
															transport vesicle	31	0.80
															membrane microdomain	33	0.76
															membrane raft	33	0.76
															supramolecular fiber	67	0.74
															polymeric cytoskeletal fiber	67	0.74

															axon	54	0.73
															axon part	29	0.72
															presynapse	31	0.72
															microtubule	38	0.71
															dendrite	59	0.70
															neuron projection	115	0.68
															cell surface	81	0.67
															neuronal cell body	58	0.67
															cell body	65	0.65
															neuron part	144	0.64
															membrane region	36	0.61
															plasma membrane part	222	0.61
															cell projection	182	0.59
															somatodendritic compartment	79	0.59
															integral component of plasma membrane	115	0.58
															cell projection part	91	0.55
															nuclear envelope	36	0.54
															intrinsic component of plasma membrane	117	0.53
															endoplasmic reticulum membrane	73	0.52
															endoplasmic reticulum	128	0.51
															nuclear outer membrane-endoplasmic reticulum membrane network	73	0.49
															non-membrane-bounded organelle	328	0.49

															intracellular non-membrane-bounded organelle	328	0.49
															Golgi apparatus part	58	0.48
															Golgi membrane	41	0.46
															endoplasmic reticulum part	79	0.45
															Golgi apparatus	101	0.41
															endomembrane system	277	0.40
															microtubule cytoskeleton	83	0.39
															protein complex	303	0.34
															cytoplasmic part	517	0.33
															cytoplasm	743	0.32
															cytoplasmic, membrane-bounded vesicle	77	0.31
															cytoplasmic vesicle	85	0.30
															extracellular region part	276	0.30
															intracellular vesicle	85	0.30
															cell periphery	406	0.27
															vesicle	250	0.27
															membrane-bounded vesicle	238	0.27
															plasma membrane	391	0.25
															extracellular region	303	0.24
															organelle part	504	0.20
															extracellular vesicle	176	0.20
															extracellular exosome	175	0.20
															extracellular organelle	176	0.19
															intracellular part	869	0.17
															macromolecular complex	326	0.16

															intracellular	896	0.16
															intracellular organelle	748	0.16
															intracellular organelle part	473	0.14
															organelle	801	0.13
															cell	1018	0.05
															cell part	1015	0.05

Table S2: Gene functional category (GFC) of up and down regulated genes for paired analysis of E2 vs Con-Ve; EPA vs Con-Ve and EPA vs E2

E2 vs Con-Ve upregulated genes			E2 vs Con-Ve downregulated genes			EPA vs Con-Ve upregulated genes			EPA vs Con-Ve downregulated genes			EPA vs E2 upregulated genes			EPA vs E2 downregulated genes		
GFC	No	FC (Log)	GFC	No	FC (Log)	GFC	No	FC (Log)	GFC	No.	FC (Log)	GFC	No.	FC (Log)	GFC	No.	FC (Log)
Thick filament*	9	6.22	Collagen degradation	5	4.33	VLDL	5	3.46	Thick filament*	10	4.05	Protein kinase inhibitor	4	2.48	Thick filament*	10	4.01
Muscle protein*	19	5.18	VLDL	3	4.22	Collagen degradation	6	3.09	Cholesterol biosynthesis	15	3.90	Iron transport	9	2.26	Cholesterol biosynthesis	15	3.85
Myosin*	11	4.51	Acute phase	5	3.78	MHC I	4	2.90	Muscle protein*	35	3.75	Collagen degradation	7	2.06	Muscle protein*	36	3.74
Sarcoplasmic reticulum*	5	3.71	Antiviral defense	18	3.62	Sushi	13	2.74	Sterol biosynthesis	16	3.62	MHC I	8	2.04	Sterol biosynthesis	16	3.57
Motor protein*	13	3.34	Complement pathway	4	3.34	Antioxidant	4	2.69	Sodium/potassium transport	4	3.32	Sushi	12	1.97	Steroid biosynthesis	18	3.19
Chloride channel	4	3.11	Innate immunity	34	3.27	Iron transport	6	2.66	Steroid biosynthesis	18	3.24	Complement pathway	8	1.85	Sarcoplasmic reticulum*	17	3.11
Basement membrane	3	3.09	Iron transport	3	3.16	Complement pathway	7	2.65	Cholesterol metabolism	24	3.12	Chemotaxis	18	1.74	Cholesterol metabolism	23	3.01
Extracellular matrix	20	3.08	Chemotaxis	12	3.13	Acute phase	6	2.54	Sterol metabolism	25	3.01	Serine esterase	6	1.74	Myosin*	19	2.93
Chloride	5	2.89	Heparin-binding	9	3.12	Peroxidase	5	2.46	Myosin*	19	2.98	Antioxidant	5	1.70	Sterol metabolism	24	2.90
Actin-binding	17	2.75	Immunity	44	2.91	Heparin-binding	16	2.45	Sarcoplasmic reticulum*	14	2.88	Neurotransmitter transport	6	1.66	Protein kinase inhibitor	5	2.78
Carbohydrate metabolism	5	2.43	Sushi	5	2.87	Gamma-carboxyglutamic acid	4	2.43	Steroid metabolism	25	2.75	Amino-acid transport	6	1.66	Steroid metabolism	24	2.64
Calmodulin-binding	7	2.32	Inflammatory response	15	2.67	HDL	4	2.43	Protein kinase inhibitor	4	2.51	Antiviral defense	23	1.58	Myogenesis*	10	2.46
Voltage-gated channel	6	2.13	Pyrrolidone carboxylic acid	5	2.54	Pyrrolidone carboxylic acid	12	2.30	CBS domain	5	2.39	Heparin-binding	16	1.46	CBS domain	5	2.34
Immunoglobulin domain	16	1.73	Serine protease inhibitor	7	2.51	Proteoglycan	9	2.28	Actin-binding	60	2.25	Copper	8	1.43	Basement membrane	9	2.31
Cell adhesion	14	1.60	Cytokine	15	2.36	Signal transduction inhibitor	7	2.08	Lipid biosynthesis	37	2.20	Inflammatory response	28	1.40	Actin-binding	64	2.29
Calcium	23	1.47	Nucleotidyltransferase	5	2.17	Biomineralization	5	2.06	Myogenesis*	8	2.19	Proteoglycan	8	1.38	Heparan sulfate	4	2.27
Secreted	41	1.28	Lipid	5	2.01	Serine	6	2.05	LIM domain	16	2.11	FAD	18	1.34	Notch signaling	11	2.17

			transport			esterase									pathway		
Disulfide bond	71	1.18	Protease inhibitor	7	1.99	Chemotaxis	16	2.04	Sodium channel	6	2.09	Pyrrolidone carboxylic acid	12	1.30	Motor protein*	29	2.13
Development al protein	22	1.16	Hydroxylation	5	1.96	Sulfation	6	2.01	Motor protein*	27	2.07	Innate immunity	54	1.28	Laminin EGF-like domain	7	2.12
Glycoprotein	82	1.10	Zymogen	11	1.82	Growth factor	21	2.00	Integrin	11	2.05	Serine protease inhibitor	10	1.22	Calmodulin-binding	29	2.01
Ion transport	13	1.06	Extracellular matrix	11	1.68	Extracellular matrix	36	1.89	Calmodulin-binding	28	2.01	Lipid transport	10	1.20	Integrin	11	2.00
Methylation	18	0.90	Microsome	6	1.64	Inflammatory response	24	1.85	Basement membrane	7	1.99	Immunity	77	1.19	Lipid biosynthesis	33	1.99
Signal	75	0.72	Secreted	73	1.57	GPI-anchor	20	1.79	Laminin EGF-like domain	6	1.95	Glycosidase	12	1.17	LIM domain	15	1.97
Nucleotide-binding	27	0.61	GTP-binding	14	1.53	Innate immunity	31	1.64	Cell shape	5	1.94	Hydroxylation	10	1.15	Mitogen	7	1.91
Coiled coil	45	0.54	Polymorphism	8	1.39	Zymogen	26	1.56	Notch signaling pathway	9	1.93	Flavoprotein	19	1.12	Cell shape	5	1.89
Cytoplasm	59	0.41	Lipoprotein	26	1.19	Lipid degradation	12	1.54	Fatty acid biosynthesis	10	1.88	Metalloprotease	18	1.11	SH3-binding	11	1.82
Phosphoprote in	91	0.25	Disulfide bond	100	1.13	Carbohydrate metabolism	11	1.53	SH3-binding	11	1.87	Lipid degradation	11	1.10	Extracellular matrix	42	1.79
			Glycoprotein	109	0.97	Lipid transport	10	1.51	Exocytosis	12	1.86	Acute phase	6	1.09	Collagen	15	1.77
			Hydrolase	46	0.94	Immunity	47	1.50	cAMP	6	1.81	Proto-oncogene	11	1.06	Exocytosis	11	1.68
			Protease	15	0.92	Flavoprotein	15	1.48	Phospholipid biosynthesis	8	1.79	Lysosome	35	1.05	Fatty acid biosynthesis	9	1.68
			Endoplasmic reticulum	27	0.89	Angiogenesis	14	1.47	Tight junction	14	1.75	Signal transduction inhibitor	8	1.05	Neurogenesis	40	1.64
			RNA-binding	16	0.87	Hydroxylation	10	1.46	Endocytosis	19	1.68	Cytokine	26	1.01	Endocytosis	19	1.63
			Signal	117	0.82	Metalloprotea se	16	1.44	Phospholipid metabolism	8	1.67	Peroxisome	11	1.01	Alternative promoter usage	9	1.58
			Nucleotide-binding	39	0.61	Secreted	188	1.43	Alternative promoter usage	9	1.63	Extracellular matrix	32	1.00	Voltage-gated channel	21	1.57
			Zinc	43	0.49	FAD	13	1.42	Signal transduction inhibitor	6	1.58	Growth factor	18	0.99	Tight junction	12	1.48
			Metal-binding	66	0.41	Cytokine	22	1.41	Voltage-gated channel	19	1.48	Sodium	12	0.97	Cell adhesion	65	1.45
			Cytoplasm	79	0.30	Collagen	9	1.36	Extracellular matrix	32	1.44	Lyase	14	0.91	Intermediate filament	10	1.36
						Symport	11	1.26	Lipid metabolism	55	1.39	Blood coagulation	7	0.90	Cell junction	84	1.29
						Microsome	13	1.25	SH3 domain	27	1.37	Zymogen	34	0.87	SH3 domain	26	1.27
						NADP	17	1.23	Neurogenesis	32	1.37	Symport	15	0.86	Angiogenesis	15	1.25
						Cleavage on pair of basic	24	1.23	Collagen	11	1.37	Secreted	201	0.85	Lipid metabolism	50	1.21

						residues											
						Monooxygenase	12	1.13	Cell adhesion	59	1.36	Peroxidase	5	0.81	Developmental protein	116	1.20
						EGF-like domain	20	1.11	Cell junction	80	1.27	GPI-anchor	18	0.79	Calcium	96	1.16
						Lysosome	22	1.10	Fatty acid metabolism	15	1.27	Protease inhibitor	14	0.76	EGF-like domain	26	1.16
						Heme	15	1.09	GTPase activation	19	1.22	Hemostasis	7	0.75	Hydroxylation	10	1.13
						Oxidoreductase	56	1.08	Sodium transport	13	1.20	NAD	20	0.71	Differentiation	73	1.12
						Serine protease	15	1.07	Angiogenesis	14	1.19	Polymorphism	24	0.69	Cleavage on pair of basic residues	28	1.12
						Neurogenesis	21	1.04	Cleavage on pair of basic residues	28	1.17	Microsome	15	0.67	GTPase activation	18	1.09
						Iron	31	1.00	Potassium transport	12	1.16	Disulfide bond	291	0.66	Cytoskeleton	116	1.06
						Disulfide bond	254	0.98	Myristate	19	1.14	Angiogenesis	16	0.63	Microtubule	28	1.03
						Glycoprotein	309	0.97	Sodium	13	1.11	Hydrolase	150	0.62	Guanine-nucleotide releasing factor	14	1.02
						Cell adhesion	35	0.88	Proto-oncogene	11	1.09	Glycoprotein	351	0.61	Fatty acid metabolism	13	1.02
						Protease	41	0.87	Calcium	88	1.08	Nucleotidyltransferase	10	0.61	NADP	18	0.99
						Signal	329	0.81	Guanine-nucleotide releasing factor	14	1.07	GTP-binding	29	0.61	Growth factor	13	0.98
						Lipid metabolism	30	0.80	Developmental protein	102	1.06	EGF-like domain	23	0.61	Wnt signaling pathway	18	0.98
						Lipoprotein	56	0.80	NADP	18	1.04	Cleavage on pair of basic residues	24	0.59	Ion channel	34	0.96
						Hydrolase	116	0.77	Microtubule	27	1.03	Lipid metabolism	32	0.58	Synapse	36	0.96
						Developmental protein	67	0.73	Potassium	12	0.99	NADP	19	0.58	Glycosyltransferase	22	0.92
						Differentiation	44	0.72	Cytoskeleton	106	0.98	Oxidoreductase	62	0.57	Kinase	67	0.87
						Calcium	56	0.71	EGF-like domain	22	0.97	Carbohydrate metabolism	11	0.56	Immunoglobulin domain	45	0.85
						Apoptosis	30	0.57	Ion channel	31	0.88	Cell adhesion	38	0.53	Signal-anchor	41	0.85
						Immunoglobulin domain	29	0.55	Lipid-binding	18	0.86	Lipoprotein	66	0.52	Postsynaptic cell membrane	16	0.81
						Metal-binding	164	0.23	Differentiation	58	0.84	Heme	17	0.51	Ion transport	55	0.78
									Synapse	32	0.84	Signal	371	0.49	Methylation	84	0.75

									Glycosyltransferase	20	0.82	Iron	33	0.48	Cell projection	57	0.70
									Signal-anchor	39	0.82	Magnesium	37	0.47	Serine/threonine-protein kinase	34	0.70
									Ion transport	53	0.77	Apoptosis	38	0.46	Coiled coil	255	0.68
									Kinase	59	0.73	Protease	47	0.43	Glycoprotein	315	0.67
									Immunoglobulin domain	39	0.69	Differentiation	44	0.41	Repressor	44	0.67
									Glycoprotein	309	0.69	Endoplasmic reticulum	70	0.39	Palmitate	25	0.67
									Lipoprotein	62	0.66	Calcium	55	0.38	Lipoprotein	64	0.66
									Methylation	76	0.66	Nucleotide-binding	108	0.27	Cytoplasm	360	0.66
									Palmitate	24	0.65	Developmental protein	67	0.26	ATP-binding	111	0.65
									Coiled coil	241	0.64	Ion transport	45	0.26	Cytoplasmic vesicle	39	0.62
									Cytoplasm	339	0.62	DNA-binding	98	0.26	Magnesium	41	0.60
									Magnesium	40	0.61	Transport	116	0.25	Golgi apparatus	59	0.60
									Cell projection	52	0.61	Metal-binding	213	0.15	Nucleotide-binding	135	0.57
									ATP-binding	102	0.58	Cytoplasm	257	0.13	Phosphoprotein	578	0.55
									Nucleotide-binding	130	0.56				Disulfide bond	237	0.55
									Serine/threonine-protein kinase	30	0.56				Alternative splicing	356	0.52
									Cytoplasmic vesicle	36	0.55				Endoplasmic reticulum	73	0.50
									Alternative splicing	349	0.54				Ubl conjugation	110	0.50
									Endoplasmic reticulum	72	0.53				Cell cycle	45	0.47
									Phosphoprotein	548	0.52				Transferase	118	0.46
									Disulfide bond	224	0.52				Activator	44	0.44
									Transferase	116	0.48				Secreted	114	0.38
									Repressor	37	0.47				Metal-binding	216	0.30
									Golgi apparatus	50	0.40				Transcription regulation	114	0.29
									Secreted	110	0.38				Transport	120	0.28
									Ubl conjugation	93	0.30				Signal	286	0.28
									Metal-binding	208	0.29				Transcription	115	0.25
									Transport	115	0.27				Cell membrane	230	0.24

									Cell membrane	226	0.26				Membrane	496	0.14
									Signal	273	0.26						
									Membrane	493	0.18						

*Genes associated with muscle structure and function

Table S3: Genes expressed in EPA and not in E2 treated cells

**Gene symbol**					
Cxcl9	Gpc4	Wnt6	Adm2	Wnt5b	Serpina3n
Prss35	Plb1	6430550D23Rik	Srgn	Gbp5	Tpsab1
Serpinb6c	Ager	Rnase1	Dlgap2	Ltc4s	Camp
Gipc2	Sectm1a	Slc16a9	Klra2	Gm5039	Serpina3g
Ang2	Snord95	Gm13483	Plek	Nos2	Hp
Apol8	Fam178b	Snord72	Kcnmb2	Mmp9	
Gm10615	Erv3	Kcna4	AW046200	Pde1a	
Tubb4a	Vsnl1	Serpini1	9430018G01Rik	C030030A07Rik	
9530053A07Rik	Ido2	Hmgcll1	Slc5a7	Gm5662	
Creb3l4	Gm6297	Gm4544	Tnxb	Gm16685	
Gm14379	BC061194	Ppm1h	Rasgef1a	Notum	
Gm4787	Atp13a4	E230016K23Rik	Birc3	Slc10a6	
Ugt1a6b	Otop1	Ttll13	Mmp8	Cd74	
Ism1	Mx1	Atp8b3	Mmp13	Epas1	
Fignl2	Gm9992	Phactr1	Trim34b	Serpina3h	
2310014F06Rik	Prl2c5	Ces1f	Apoc1	Orm1	
Ces1d	AA467197	Gsta2	Dub1	Neurl3	
AW549542	Zfp33b	G630090E17Rik	Cd33	Il6	
2810454H06Rik	Oas1c	Tnip3	Orm3	Slfn2	
Pacrg	Gm1661	Akr1c12	Ncf4	Steap4	
Gm11517	Cd46	Car6	Gm2022	Gm11428	
P2rx3	Dennd2d	Tgfa	AI747448	Lcn2	
Fbxw10	Gm20045	A530020G20Rik	Lypd6b	Atp8b4	
C430002N11Rik	Hmha1	Ces1g	Serpina3i	Mmp3	
Stard6	Hlf	Slc12a5	Mpeg1	Tnfrsf9	

Table S4: Genes expressed in E2 but not in EPA treated cells.

Gzmd	Nudt15	Palmd	Gm5532		
Dscam	Myoz1	Aim1l	Arx	Igsf5	Lrrc14b
Wnt7b	Kcnh7	Gm10421	Klrb1a	Gpd1	Tekt5
Gm19383	Ramp1	Ankmy1	Gm17638	Limch1	Shisa2
Fes	Gm7889	Adamts16	LOC100862154	2410076I21Rik	Hspb3
Csn3	Padi3	Pthlh	Ripply1	Sprr2k	Rbfox1
Klk10	Syt7	9430070O13Rik	Chst13	Bzrap1	Il2rb
Rasgrp3	Pacsin1	Nrarp	Art5	Clcnka	C130080G10Rik
Tceal5	Dok3	Fam78a	Nup210	F2rl1	Camk2b
Tdrd9	S100a14	Cd163	Ysk4	Fsd2	Fras1
Mpp3	Themis	Upk1b	Atp6v1c2	F2	Gpr20
Hoxc12	Odf3l1	B3galt2	I830127L07Rik	Cnn1	Gm16534
Gpr37	Cpa4	Krt31	Gm10494	Plxna4	Mybpc2
Lamc3	Slco5a1	Emid2	St8sia2	1810041L15Rik	B3galt5
Pof1b	Gm19976	Chi3l1	Pard6b	Gm19494	4833422C13Rik
F2rl2	Grb10	Ampd1	Crabp1	Adcy8	Fgfbp1
Ptpn7	Neu2	Gzmm	Tnni3	Tspan8	Syt8
Gm14230	Otoa	Clrn1	Tcf23	Dsg3	Gbp8
Efcab5	Flrt1	Traf1	Ctsw	Sohlh2	Dnase1l3
Lrrc4	Nfe2	Gpr113	Prox1	Grip2	Fmod
Fut1	Ptgds	1500015O10Rik	Grhl3	Art3	Padi2
Nwd1	Adamts17	Cplx3	Gbp11	Egflam	Gja5
Plac1	Best3	Cacng6	Agmat	Myh14	Tspan33
Jakmip1	Asb16	Tex16	Isx	LOC100861990	
	Cyp11a1	Cdh17	Mafa	Cldn2	
